# Self-organized flexible leadership promotes collective intelligence in human groups

**DOI:** 10.1098/rsos.150222

**Published:** 2015-12-23

**Authors:** Ralf H. J. M. Kurvers, Max Wolf, Marc Naguib, Jens Krause

**Affiliations:** 1 Center for Adaptive Rationality, Max Planck Institute for Human Development, Lentzeallee 94, Berlin 14195, Germany; 2 Department of Biology and Ecology of Fishes, Leibniz-Institute of Freshwater Ecology and Inland Fisheries, Müggelseedamm 310, Berlin 12587, Germany; 3 Behavioural Ecology Group, Department of Animal Sciences, Wageningen University, De Elst 1, Wageningen 6708 WD, The Netherlands; 4 Department of Crop and Animal Sciences, Humboldt-University of Berlin, Berlin, Germany

**Keywords:** swarm intelligence, collective decision-making, groups, information, leadership

## Abstract

Collective intelligence refers to the ability of groups to outperform individual decision-makers. At present, relatively little is known about the mechanisms promoting collective intelligence in natural systems. We here test a novel mechanism generating collective intelligence: self-organization according to information quality. We tested this mechanism by performing simulated predator detection experiments using human groups. By continuously tracking the personal information of all members prior to collective decisions, we found that individuals adjusted their response time during collective decisions to the accuracy of their personal information. When individuals possessed accurate personal information, they decided quickly during collective decisions providing accurate information to the other group members. By contrast, when individuals had inaccurate personal information, they waited longer, allowing them to use social information before making a decision. Individuals deciding late during collective decisions had an increased probability of changing their decision leading to increased collective accuracy. Our results thus show that groups can self-organize according to the information accuracy of their members, thereby promoting collective intelligence. Interestingly, we find that individuals flexibly acted both as leader and as follower depending on the quality of their personal information at any particular point in time.

## 1. Introduction

Collective intelligence refers to the ability of groups to make better decisions than single individuals when solving cognitive problems [1–4] and can be found in a wide variety of organisms such as microbes [5], insects [6], fish [7] and humans [1,8–10]. Understanding the mechanisms generating collective intelligence is crucial for both our understanding of animal organizations and the design of human decision-making systems. Most of the currently known collective intelligence mechanisms are based on aggregating the opinions of many individuals (e.g. information pooling, averaging and quorum sensing) whereby increased collective accuracy is achieved by combining imperfect individual estimates [8,11–16]. Here we test a novel mechanism by which collective intelligence may arise: self-organization according to information quality. In a nutshell, this mechanism predicts that individuals base their contribution to a collective decision on the quality of their personal information. In particular, whenever individuals have high-quality personal information, they take the lead in collective decisions, whereas when the quality of their personal information is poor they follow. As a consequence, whenever individuals differ in the quality of information over repeated decision-making events, this behaviour would allow groups to base their decisions on the best information available at any point in time and thus (over repeated decision-making events) outperform single decision-makers.

In line with our hypothesis, several empirical studies have shown that providing individuals with information can turn them into leaders [17–23]. However, most of these studies trained individuals into ‘experts’ that are inherently better decision-makers than the other group members (though some studies used the natural inter-individual variation in learning abilities [21] or stochastic differences in individual exploratory behaviour [20]). While such experts certainly exist in some situations (e.g. more experienced and/or older group members [24–26]), in many other situations the individuals that have superior information change over time, depending on, for example, context-dependent abilities and experience, time investment and orientation. The question then arises how individuals in groups make decisions over repeated decision-making events when the individuals that have high-quality information continuously differ and when it is unclear which individuals possess high-quality information at any given time? We here propose that the above hypothesized mechanism (i.e. reduce the contribution to collective decision when uncertain) can provide a solution for such situations.

To test this mechanism, we presented human groups with a simulated predator detection task. Human groups briefly saw an image of a group of animals that sometimes contained a critical amount of predators and sometimes not (electronic supplementary material, figure S1). Individuals first decided by themselves using wireless voting machines whether they wanted to ‘stay’ or ‘escape’ (providing us with their personal information). Then there was a collective decision regarding the same image, in which decisions that individuals made were immediately visible to their group members. Individuals could thus decide early thereby potentially influencing others or wait for social information. Each group was tested a large number of times, whereby we always kept track of the personal information of all individual members prior to each collective decision. During all decision-making events the software registered how quickly each individual made a decision and from this we calculated the order of decision-making during all decision sequences. This allowed us to test whether groups self-organized according to information status of the individual agents.

## 2. Methods

We recruited 219 students (from Wageningen University, The Netherlands) spread over 11 groups (range of group sizes: 17–23; May 2012 and May 2014). Participants were second and third year students following a course on behavioural ecology at the Wageningen University. Prior to the experiments we obtained informed consent from all participants. All experiments were done in accordance with the principles of the Declaration of Helsinki. Each group was confronted with a simulated predator-detection experiment. Individuals belonging to the same group entered the test room together and individuals were seated on chairs (next to each other) facing a white screen. In the test, participants saw for 2 s a school of 72 fish (aligned in an 8×9 grid) containing two types of fish: ‘tuna’ and ‘sharks’ (electronic supplementary material, figure S1), projected on the white screen. Participants were instructed to adopt the following rule ‘If there are four or fewer sharks then it is safe and you should stay, if there are five or more sharks then it is too dangerous and you should escape’. After 2 s of looking at a school of fish, the participants had 5 s to take an individual decision, using electronic keypads (Reply mini+ Keypads, Fleetwood Electronics) to indicate their decision. Participants were instructed to press a ‘1’ for ‘stay’ and a ‘2’ for ‘escape’. Individuals were not allowed to communicate with each other to assure independent decisions. The keypads were connected to a receiver station in a laptop which registered all decisions and response times (i.e. how quickly decisions were made) of all individuals (Key Point Interactive Audience Software for Power Point, version: 2.0.142 Standard Edition). After all individuals provided their individual decision (hereafter called polling 1), participants were asked to make a second decision (polling 2) regarding the same fish group, but without seeing the image again. During this collective decision, individuals were again asked to press ‘1’ for ‘stay’ and ‘2’ for ‘escape’. This time, however, whenever an individual made a decision, the decision became immediately visible to the other group members by means of two bar charts indicating the number of people who indicated ‘stay’ and ‘escape’. Public information became thus gradually available and individuals could choose to press early with the potential of influencing other group members, or wait for social information. Individuals could decide only once during polling 2 (i.e. could not alter their decision) and had 12 s to make a decision. Afterwards, we showed the correct answer after which a new round started. During the polling periods, there was a countdown timer visible at the screen and a music tune playing to indicate the polling period. Two individuals were excluded from all analyses because they missed a large fraction of the polling periods (more than 30%). The remaining 217 individuals only occasionally (≈2%) missed the polling period. These missing values were also excluded from all analyses.

There were four different treatments: three, four, six or seven sharks (and 69, 68, 66 and 65 tuna, respectively), and each treatment was replicated seven times resulting in a total of 28 rounds (with each round consisting of two polling moments). The treatment order and the position of the sharks in the school of fish were randomized. Prior to the 28 rounds, we performed two test rounds (not included in the analysis) to instruct the participants about the procedure. Each of the 11 groups completed all the 28 rounds. Note that a correct decision is made if a participant ‘stays’ in the presence of three or four sharks and ‘escapes’ in the presence of six or seven sharks. Individuals belonging to the best performing group (within each year) received a small reward (chocolate bars) after all groups completed the experiment.

The software registered when each individual made his/her decision. From these response times, we calculated for each decision sequence the decision order. For each group, for each round and for each polling period, we ranked the individuals according to their response time and the individual with the lowest response time received a decision order of ‘1’, the second lowest ‘2’, etc. These values were then divided by the group size to facilitate a comparison between the 11 groups. This ‘response order’ (range: 0.043–1) thus indicates the order of decision-making with low values indicating early deciders and high values late deciders. We used decision order rather than decision time since order is more informative with regard to information availability (e.g. a decision time of 5 s could mean that there is no information available yet, since everybody is waiting, or it could be that there is ample social information since most group members already made a decision. By contrast, a decision order of ‘0.5’ implies that for a group size of 20, that individual is the 10th person to make a decision, making this a more objective measure of social information availability).

## 3. Statistical analysis

To test whether individuals made better decisions during polling 2 than during polling 1, we calculated the average accuracy rate (i.e. the fraction of correct decisions) per individual in both polling periods. These average accuracy rates were then used as the response variable in a generalized linear mixed model (GLMM). As fixed effect we fitted polling (1 or 2) and as random effect individual nested in group.

To test whether individual information accuracy affected the order of decision-making during polling 2, we determined for each individual its average decision order during polling 2 for (i) all rounds in which the individual decision (i.e. polling 1) was correct and (ii) all rounds in which the individual decision was wrong. This variable was used as a response variable in a GLMM to test the effect of ‘individual decision correct’ (fixed effect) on decision order in the subsequent polling 2. Individual nested in group was used as a random effect. A positive effect of ‘individual decision correct’ on decision order during polling 2 would imply that individuals wait longer with making a decision during polling 2 if they made a wrong decision during the preceding polling 1 (i.e. have inaccurate personal information).

To test whether decision order during polling 2 affected the probability that an individual changed its opinion between polling 1 and 2 within that round, we determined for each individual for each round whether the individual changed its opinion between polling 1 and 2 (yes/no). This variable was used as the response variable in a GLMM. As fixed effect, we fitted the decision order during polling 2. As random effect, we used individual nested in group and round nested in group. Note that this analysis is not done with average values on the level of individuals but on the level of each round. Last, we tested if there was an effect of decision order during polling 2 on the probability of making the correct decisions during polling 2 using a similar GLMM procedure.

In principle, even random decision sequences during polling 2 (i.e. a decision sequence that is independent of the quality/correctness of information) could improve collective decisions, especially when a large majority of the respondents are already correct in polling 1. To test whether the orderliness of the decision-making sequence during polling 2 *per se* positively affected collective improvements, we calculated per round the collective improvement (defined as accuracy rate polling 2 – accuracy rate polling 1) and the average decision order of individuals during polling 2 who were wrong during the preceding polling 1. We used this latter variable as a proxy for the orderliness of the decision-making sequence: for any particular round, the higher the average decision order of individuals in polling 2 who were wrong during the preceding polling 1, the higher the degree of orderliness of the decision-making sequence in that round. This variable (average decision order of individuals during polling 2 who were wrong during the preceding polling 1) was then used as a fixed effect in a GLMM with collective improvement as response variable. Group was fitted as random term. Under our scenario of ‘self-organization based on information quality’, we expected that collective improvements would be the highest when individuals who made wrong decisions during polling 1 decide late during polling 2. For this analysis, we only included rounds in which at least 20% of decision-makers were wrong during polling 1 since in the other rounds there were only very few individuals who could improve.

To test whether individual quality *per se* affected the decision order during polling 2, we calculated per individual the average order of decision-making during polling 2. This variable was then used as a response variable in a GLMM. As fixed effect, we fitted the average individual accuracy rate during polling 1 (as a measure of individual quality) and group was fitted as a random effect. A negative effect of individual accuracy rate on decision order in polling 2 would imply that individuals with a high individual accuracy rate are consistently early deciders in polling 2 (i.e. consistent leadership according to performance).

For all GLMMs, we used binomial errors and a logit-link function since all response variables were either rates (bound between 0 and 1) or binary (0 or 1). For the GLMMs, we used the package lme4 for mixed model procedures in R (v. 3.1.1, R Development Core Team 2011). Significance levels of individual factors were derived from the *z*-values and associated *p*-values. These are reported, as well as the estimates and standard errors of individual factors unless stated otherwise.

Furthermore, we analysed our data separately for the treatments with three or four sharks and treatments with six or seven sharks since we might expect differences between these two scenarios. Whenever there are six or seven sharks present, individuals have the opportunity to count all of them and be completely certain about their decision. However, in the case of three or four sharks, even counting all sharks cannot completely reduce uncertainty, since individuals cannot exclude the possibility that they might have missed some sharks. Therefore, a stronger effect of leadership according to information quality might be expected in the treatments with six or seven sharks.

## 4. Results

**Figure 1.**
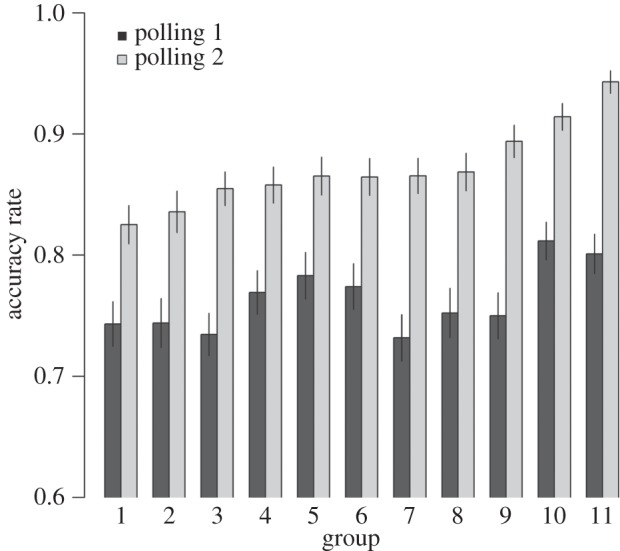
Accuracy rates per group during polling 1 (dark grey bars) and polling 2 (light grey bars). The accuracy rate of all groups was higher in polling 2 than in polling 1. Shown are means ± 95% CI. For clarity, groups are arranged according to their accuracy rate in polling 2.

Individuals made better decisions during polling 2 than during polling 1 (est ± s.e. = 0.76 ± 0.26, *z*=2.9, *p*=0.003) and this pattern was very consistent across groups: in all 11 groups the decision accuracy was higher in polling 2 than in polling 1 (figure 1). This improvement was also evident on the level of the individual when comparing the accuracy rate during polling 1 and 2 per individual. Out of the 217 individuals, 182 individuals (83.9%) made better decisions during polling 2 than during polling 1, 23 individuals (10.6%) had equal accuracy during polling 1 and 2, and only 12 individuals (5.5%) made worse decisions during polling 2 (electronic supplementary material, figure S2). The large majority of individuals thus made better decisions during polling 2. Both in the rounds with three or four sharks and in the rounds with six or seven sharks, decision accuracy was higher in polling 2 than in polling 1 (electronic supplementary material, figure S5).

**Figure 2.**
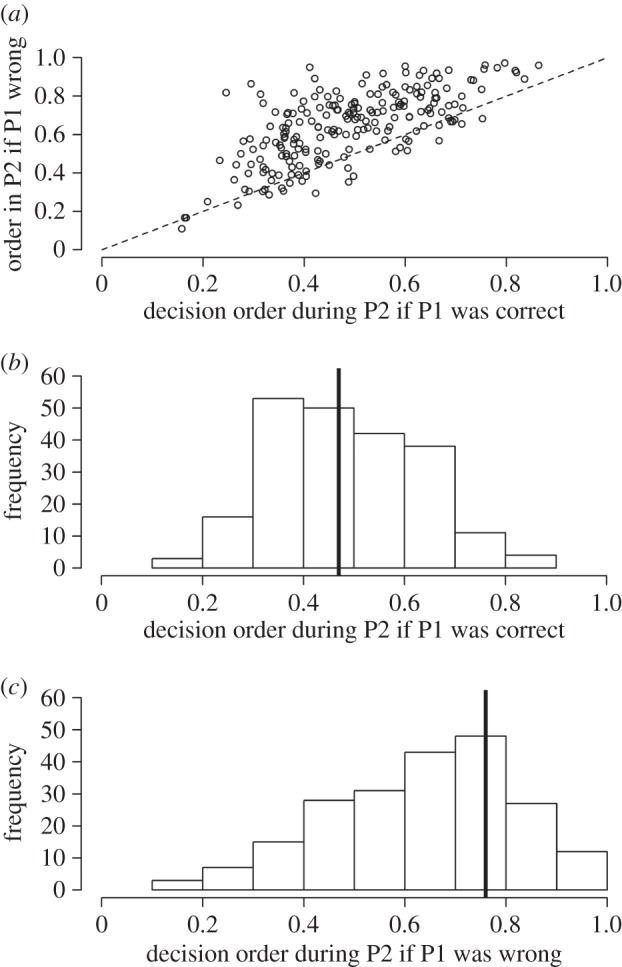
(*a*) The relationship between an individual’s average decision order during polling 2 when its preceding polling 1 was correct and when its preceding polling 1 was wrong. The dotted line shows the situation in which there is no effect of ‘individual decision correct (y/n)’ on decision order in the subsequent polling 2. The majority of individuals are above this predicted line, implying that individuals decided later during polling 2 if they made a wrong decision during the preceding polling 1 than when they made a correct decision during the preceding polling 1. (*b,c*) Histograms show the frequency distribution of the average decision order per individual during polling 2 (*b*) when the decision during polling 1 was correct and (*c*) when the decision during polling 1 was wrong. Black lines represent medians. P1, polling 1; P2, polling 2.

**Figure 3.**
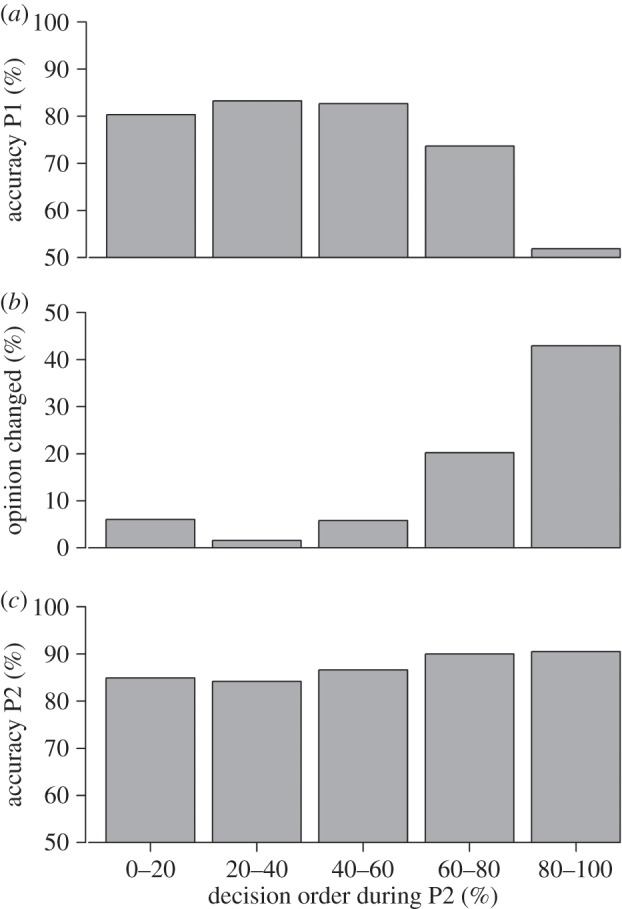
Individuals that decided later during polling 2 were (*a*) more likely to be wrong during the preceding polling 1 and (*b*) more likely to change their opinion comparing polling 1 and polling 2. As a result, (*c*) individuals that decided later during polling 2 were more likely to be correct during polling 2. For illustrative purpose, the decision order during polling 2 (*x*-axis) is shown in five groups (first 20% of decision-makers, 20–40%, 40–60%, 60–80% and last 20%). P1, polling 1; P2, polling 2.

How did this improvement arise? Because we knew the personal information of each group member prior to each collective decision and the order of decision-making during each decision sequence, we were able to test whether individuals adjusted their decision order as a function of the quality of their personal information. We found a strong effect of ‘individual decision correct (y/n)’ on the decision order in the subsequent polling 2 (est±s.e.=−0.65±0.20, *z*=−3.29, *p*=0.001; figure 2*a*). Individuals decided earlier during polling 2 when they made a correct decision during the preceding polling 1 (figure 2*b*, median: 0.47) than when they made a wrong decision during the preceding polling 1 (figure 2*c*, median: 0.76; see also figure 3*a*). This was true for the vast majority of individuals (85%) and this effect was present in rounds with three or four sharks as well as in rounds with six or seven sharks (electronic supplementary material, figure S6). There was no significant effect of ‘individual decision correct (y/n)’ on the decision order during polling 1, although there was a negative trend (est±s.e.=−0.33±0.19, *z*=−1.72, *p*=0.09; electronic supplementary material, figure S3). Individuals thus only tended to decide earlier or later in polling 1 depending on whether they made a correct or wrong decision during this polling 1. This pattern only emerged as being significant in polling 2 where individuals could strategically wait for social information.

So whenever individuals had poor personal information they decided later during polling 2. How did this affect collective accuracy? To test this, we studied how the decision order during polling 2 affected the probability that individuals changed their decision between pollings 1 and 2. Individuals that decided later during polling 2 had a higher probability of changing their decision (est±s.e.=5.16±0.20, *z*=25.70, *p*<0.001; figure 3*b*). To illustrate, of the first 20% of decisions taken during polling 2 only 6% changed, whereas for the last 20% of decisions taken during polling 2 43% changed (figure 3*b*). Almost all of these changes (90.7%) were towards the correct answer (cf. figure 3*a*,*c*). Individuals that decided later during polling 2 also had a higher probability of being correct in that collective decision (est±s.e.=0.8±0.16, *z*=5.06, *p*<0.001; figure 3*c*). Again, these effects were present in rounds with three or four sharks and in rounds with six or seven sharks (electronic supplementary material, figure S7).

**Figure 4.**
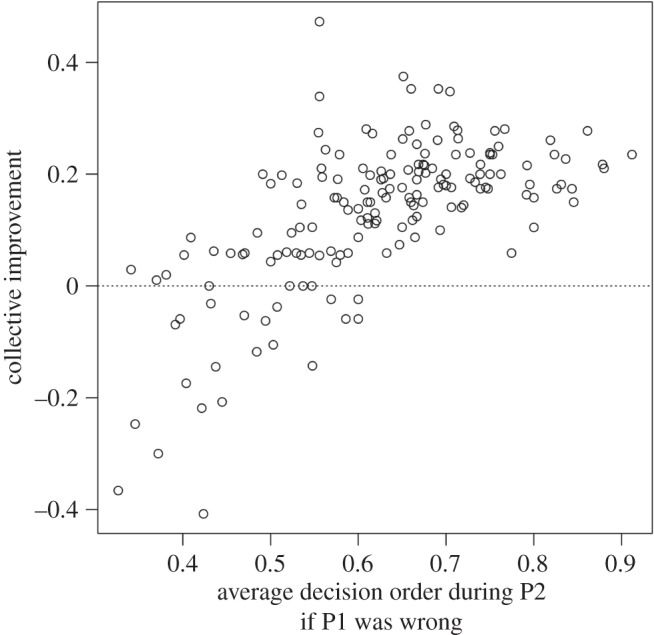
The relationship between the average decision order during polling 2 for individuals who were wrong during the preceding polling 1 and the collective improvement (accuracy rate polling 2 – accuracy rate polling 1). Each dot represents one round. Collective improvements arose when individuals who were wrong during polling 1 decided late during the subsequent polling 2. When individuals who were wrong during polling 1 decided early, collective decisions could even become worse, though this happened relatively rarely.

As discussed above, in principle, even random decision sequences in polling 2 could improve collective decisions. However, the later the individuals who were wrong during polling 1 decided during the subsequent polling 2, the higher the collective improvement (est±s.e.=2.90±1.29, *z*=2.25, *p*=0.025; figure 4; electronic supplementary material, figure S8), illustrating that the observed collective intelligence effect is truly a result of the self-organization of the groups and not merely a result of the availability of social information. In fact, as can be seen in figure 4, when individuals who were wrong during polling 1 decided relatively early during the subsequent polling 2, collective decisions could even deteriorate.

An alternative mechanism to ‘self-organization according to information quality’ is the presence of consistent leaders and followers. We, for example, might expect that there are substantial differences in individual performance that affect the decision order during collective decisions. Under this scenario, high individual performers are expected to consistently decide early during collective decisions. We tested whether individual quality *per se* affected the average decision order during polling 2 by calculating the performance of each individual during polling 1 and its average decision order during polling 2. Individual average performance ranged from 0.48 to 1 (electronic supplementary material, figure S4), but there was no significant effect of the average performance during polling 1 on the average decision order during polling 2 (est±s.e.=−1.65±1.23, *z*=−1.34, *p*=0.18; electronic supplementary material, figure S4), suggesting that individual quality *per se* did not affect the order of decision-making in polling 2.

## 5. Discussion

Individuals adjusted their response time during the collective decisions to the accuracy of their personal information. Individuals waited longer during the collective decision if they had made an incorrect decision individually than when they individually had made the correct decision (i.e. lower uncertainty). Moreover, individuals that decided later during the collective decision were more likely to change their opinion. Individuals thus self-organized according to information accuracy promoting collective intelligence.

Collective improvements depended critically on the orderliness of the decision sequence during polling 2: the later those individuals that were wrong during polling 1 decided in polling 2, the higher the collective improvement in polling 2. Interestingly, when individuals who were wrong during polling 1 decided relatively early in polling 2, collective decisions could even deteriorate. Only in polling 2, there was a significant effect of ‘individual decision correct (y/n)’ on decision order (figure 2*a*). This relationship was not significant in polling 1 (electronic supplementary material, figure S3). However, individuals also had more time to make a decision during polling 2 than during polling 1 (12 versus 5 s), which could also facilitate the orderliness of the decision sequence.

When looking in detail at those individuals that changed their decision (figure 3*b*), we see that the large majority of changes happened in the last 60–100% of decisions being made during the collective decision. The first 60% of decision-makers hardly ever changed their decision. This might be because (i) early deciders tended to have better information (i.e. were more often correct in polling 1) and (ii) during later stages of the collective decision process more information was available than during earlier stages. The vast majority of participants made better decisions with social information than without social information, suggesting that this is an adaptive use of social information [27–29]. Only a small minority made worse decisions during polling 2 than during polling 1. Possibly, these were high performing individuals that were occasionally led in the wrong direction during polling 2 because of wrong information coming from other group members.

Whereas we directly focused on the effects of correct versus wrong individual decisions on subsequent behaviour during collective decisions, more mechanistically, the decision speed during polling 2 might often be affected by the confidence individuals have in their decision during polling 1. Here we expected an asymmetry between rounds with three or four sharks and rounds with six or seven sharks because in the latter, individuals had the opportunity to count all sharks thereby minimizing uncertainty. Individuals who thus were individually correct in these situations could have potentially been more confident than individuals being individually correct in the presence of three or four sharks. The pattern of self-organization according to information quality did seem to be slightly stronger in the treatments with six or seven sharks as compared to treatments with three or four sharks (electronic supplementary material, figures S5–S8).

Several recent studies have highlighted negative effects of social influence [30,31] and the risk of false information cascades in groups [32,33]. Here, in contrast, we show that social influence can be beneficial and that sequential decision-making allows for increased accuracy. A critical prerequisite for this is that individuals need to be able to correctly assess the certainty of their personal information. Under this condition, individuals can positively influence the decision of other group members by deciding quickly (as in our experiment) or, for example, by expressing their confidence level to others [34,35]. Conversely, if individuals misjudge their own ability (e.g. [36]), then flexible leadership could have detrimental effects on collective accuracy. There is a growing interest in combining independent opinions to achieve higher collective accuracy, for example, in predictions markets [37,38]. An important consideration when combining decisions is whether all opinions should be weighted equally or weighted according to previous performance profiles of individuals. Here we show that, although there is considerable variation in individual performance (electronic supplementary material, figure S4), it is actually difficult to predict which individual possesses high-quality information at any time. So, in these contexts, rather than creating profiles based on previous performances, it is more important to have information on the confidence level of individuals for each prediction. However, in other contexts, for example, decision problems that can only be solved by a select group of experts that could be identified based on track records in solving similar problems (e.g. [39]), our approach would most likely not be applicable.

While we focused on an experimental set-up where individuals were provided with the exact same information, flexible leadership depending on information status is probably even more pronounced under natural conditions in which individuals differ in their position and orientation in the group and thus receive different information about the environment. Berdahl *et al.* [16] showed that larger fish schools were better at sensing the environment by selecting the (preferred) darker areas in a complex light environment. Individuals adjusted their swimming speed to the local light conditions (i.e. slowed down when dark, sped up when light), and by a combination of individual adjustments and social interactions, larger groups were better at avoiding the light. Though determining the personal information of individuals in groups is a continuous challenge, sophisticated tracking algorithms [40] combined with tracking the sensory input of individuals [18] can facilitate a detailed understanding of information flow in groups.

Our results suggest that individual differences in cognitive performance (e.g. the ability to detect a predator) [41] can be difficult to detect during collective decision-making since these differences are outweighed by information differences between individuals in groups (see also [42]). In a wide variety of species consistent individual differences in cognitive abilities have been documented (e.g. [43]), but whether and how these differences, in turn, affect how much individuals contribute to collective decisions is largely unknown (see also [44]). From an optimality perspective, it is expected that individuals with high cognitive performance (and in the absence of any conflicting preferences) will contribute more to collective decisions, but this remains largely untested. In homing pigeons, there was only little evidence that the individual with the highest navigational performance in a pair contributed more to leadership [45]. Next to differences in performance, there are several other factors that can play a role in contributing to leadership [46]. Recent years has seen the emergence of the field of animal personality [47,48] and several studies have highlighted how bolder individuals can exert a greater influence on group movements in animals [49–52]. In humans, extraversion has been linked to leadership [53,54] and inter-individual differences in social information use have been found in humans [55]. Next to personality, also dominance has been shown to affect leadership [56]. Our experimental set-up allowed anonymous information exchange between the interacting agents which most probably downplayed a potential role of personality and/or dominance (as compared to more direct ways of interactions, e.g. discussions or movement [57]).

Future experiments could investigate in more detail the relationship between speed of decision-making of individuals in groups and their information accuracy, to study how widespread our decision-making mechanism is and under which environmental conditions it can be found. Generally speaking, when early deciders have high-quality information, a collective benefit is expected. Conversely, if early deciders have low-quality information, there is a risk of false information cascades [32]. In our study, we used a very small reward based on group performance. Future studies could also investigate the effects of different reward structures on the collective dynamics. In escaping predation, for example, individuals that detect a predator early and react quickly have a larger probability of escaping than slow responders. Such competition could be easily incorporated by speed-dependent rewards to investigate the consequences for the collective dynamics [58]. Also, while we used an anonymous way of exchanging information, in many situations factors like personal experience or reputation of interaction partners might alter information use [59,60]. Finally, in earlier work we showed that presenting all decisions of polling 1 simultaneously also improves decision accuracy [14,61]. Future studies could investigate how the structure of information exchange interacts with decision-making context/task, and which mechanism should be used in which environment.

To conclude, we showed that human groups can self-organize in such a way that informed individuals take the lead dependent on their information accuracy. This process resulted, in the absence of any clear leaders or followers, in an improvement in collective decision accuracy.

## Supplementary Material

ESM Supplementary Data

## Supplementary Material

ESM Supplementary Information

## Data accessibility

The dataset supporting this article has been uploaded as part of the electronic supplementary material.
