# Supplementary material for: Self-organized flexible leadership promotes collective intelligence in human groups
Source: R Soc Open Sci. 2015 Dec 23;2(12):150222. doi: 10.1098/rsos.150222 (PMC4807439; doi:10.1098/rsos.150222)

**Supplemental Information**

**Self-organized flexible leadership promotes collective intelligence in human groups**

Ralf HJM Kurvers, Max Wolf, Marc Naguib & Jens Krause

**Figure S1:** Screenshot of one school of fish shown to the participants. This particular group contains four sharks and 68 tuna. All other schools shown were arranged similarly (aligned in a square shape of 8 x 9 fish), but differed in the number and position of the two types of fish. Sharks are indicated by different shape and larger fins.


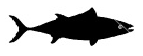

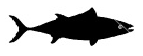

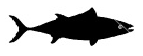

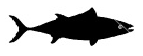

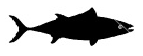

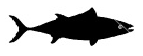

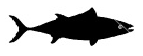

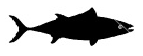

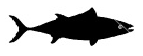

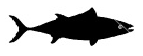

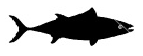

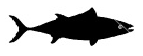

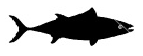

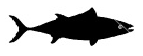

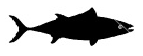

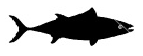

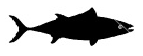

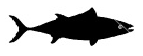

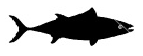

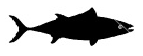

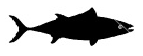

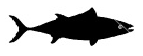

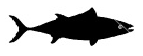

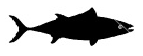

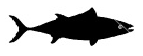

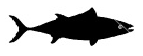

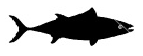

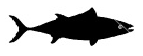

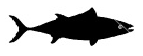

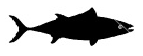

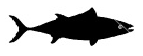

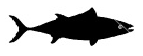

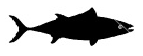

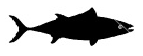

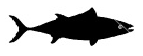

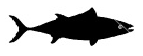

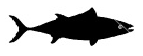

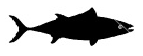

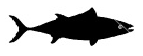

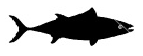

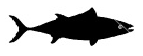

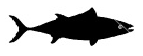

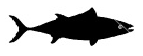

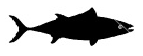

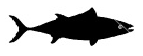

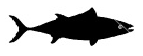

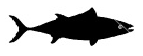

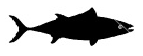

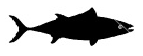

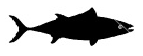

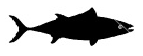

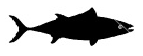

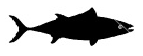

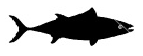

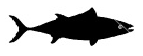

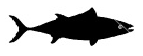

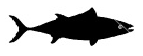

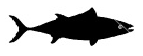

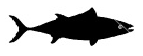

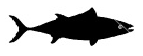

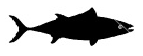

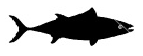

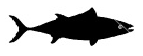

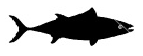

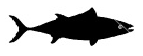

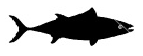

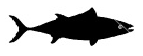

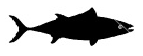

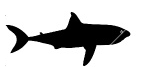

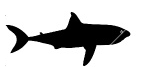

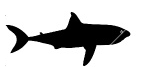

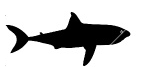


**Figure S2:** Histogram showing the frequency distribution of individual improvements in accuracy rate comparing polling 1 and 2. Most individuals (83.9%) made better decision during polling 2 than during polling 1. The median improvement (black line) equals 0.107, which corresponds to an increase in accuracy rate from, for example, 0.7 to 0.807.


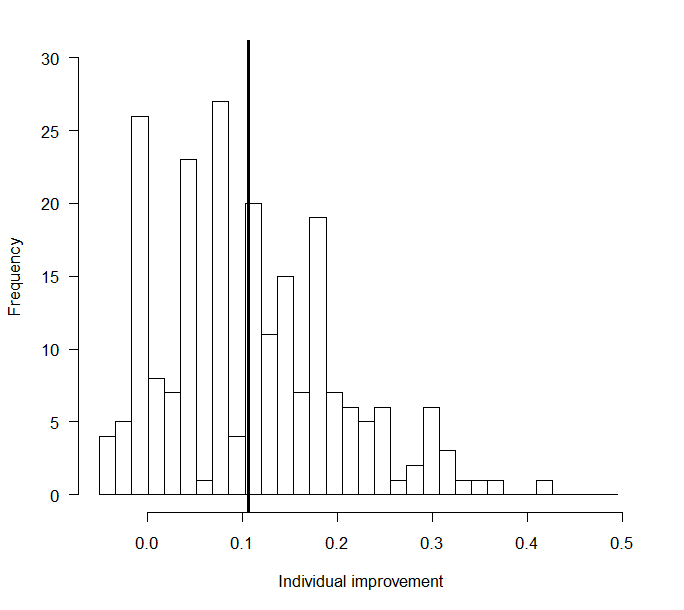


**Figure S3:** The relationship between an individual’s average decision order during polling 1 when their decision in polling 1 was correct, and when their decision in polling 1 was wrong. The line shows the prediction that there is no effect of ‘individual decision correct (y/n)’ on the decision order in polling 1. There was no significant effect of ‘individual decision correct (y/n)’ on the decision order during polling 1, although there was a negative trend (est ± SE = -0.33 ± 0.19, z = -1.72, p = 0.09).


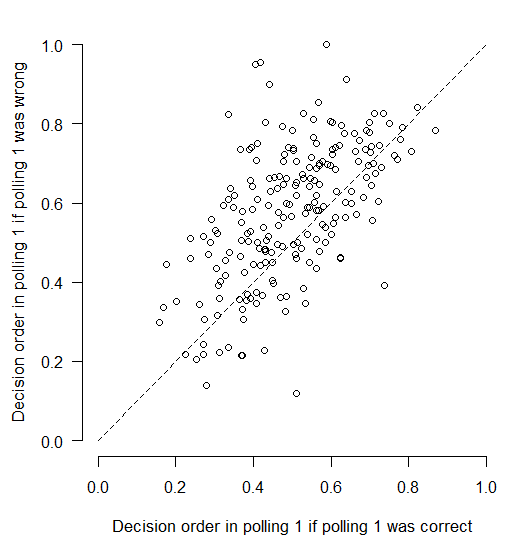


**Figure S4:** There was no effect of average individual accuracy rate during polling 1 on the average order of decision making during polling 2.


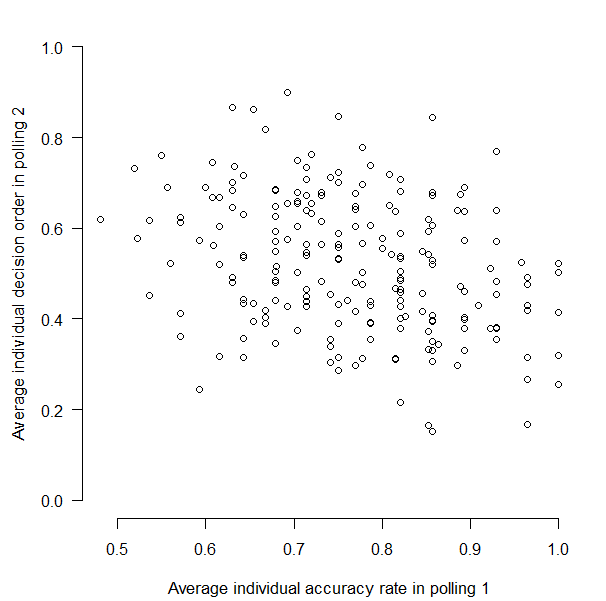


**Figure S5:** Both in treatments with 3 or 4 sharks present and 6 or 7 sharks present, individuals made better decisions in polling 2 than in polling 1.


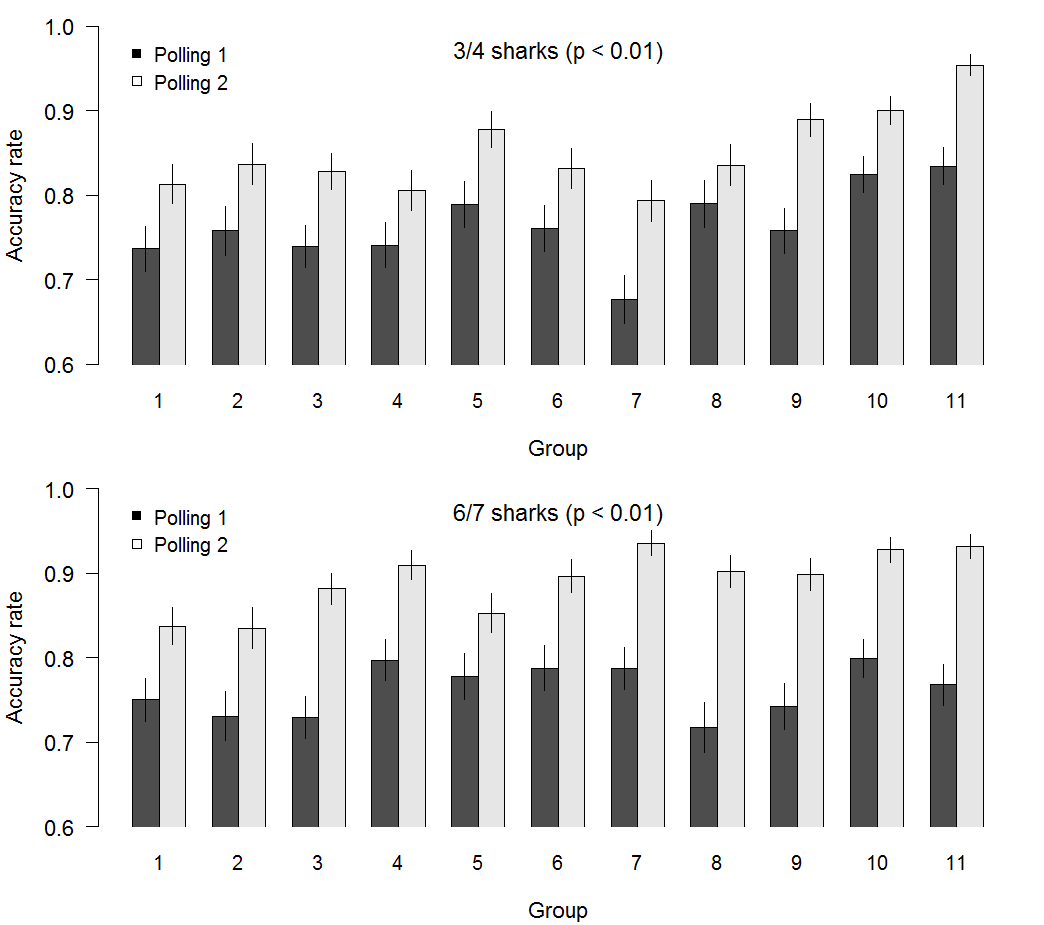


**Figure S6:** Both in treatments with 3 or 4 sharks present and 6 or 7 sharks present, individuals made faster decision during polling 2 when they made a correct decision during the preceding polling 1, than when they made a wrong decision during the preceding polling 1.


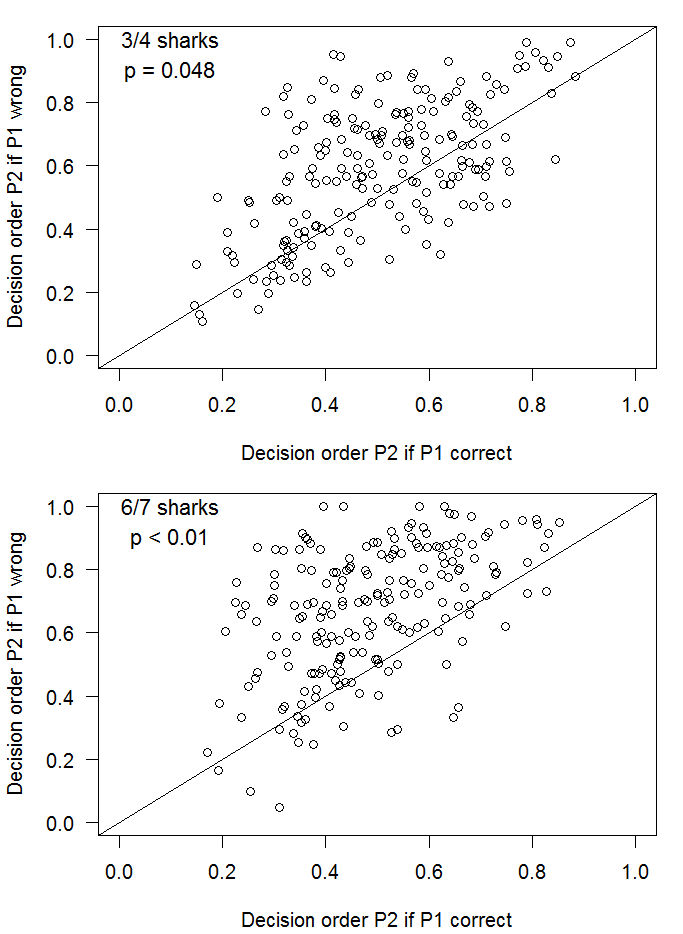


**Figure S7:** Both in treatments with 3 or 4 sharks present and 6 or 7 sharks present, individuals that decided later during polling 2 were (a) more likely to be wrong during the preceding polling 1 and (b) more likely to change their decision comparing polling 1 and 2. (c) Only in the treatment with 3 or 4 sharks there was a positive relationship between decision order during polling 2 and accuracy in polling 2.


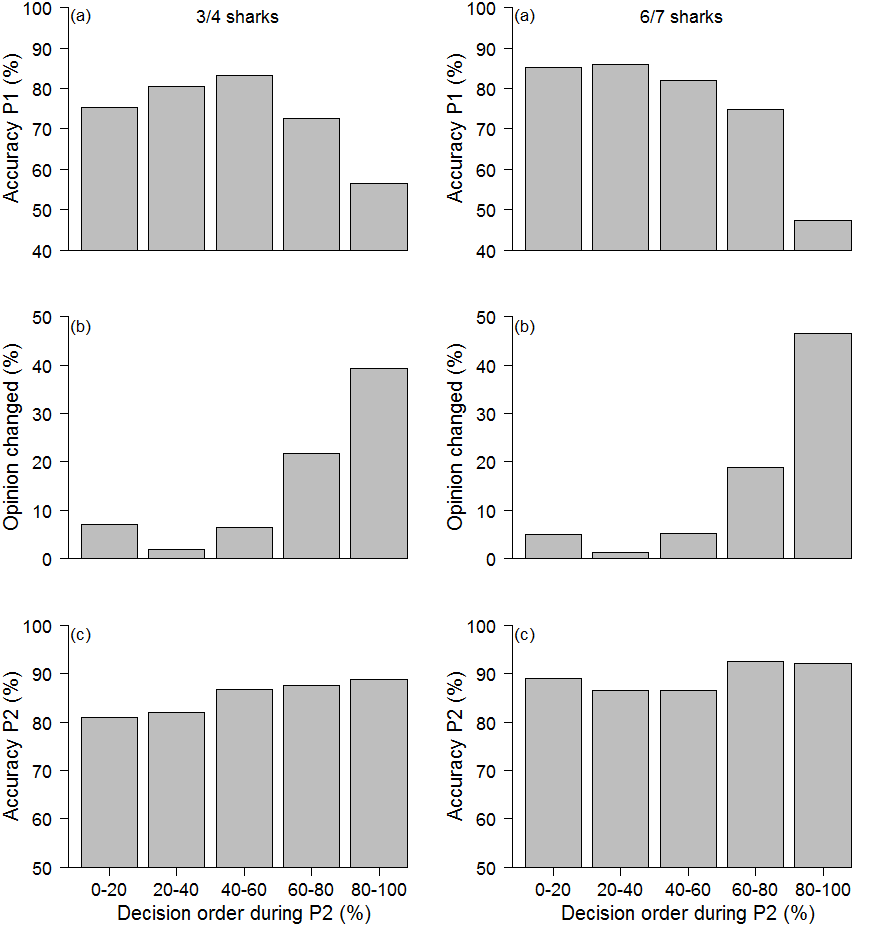


**Figure S8:** The relationship between the average decision order during polling 2 for individuals who were wrong during the preceding polling 1 and the collective improvement (accuracy rate polling 2 - accuracy rate polling 1). Each dot represents one round. Data are shown separately for treatments with 3 or 4 sharks present, and treatments with 6 or 7 sharks present.


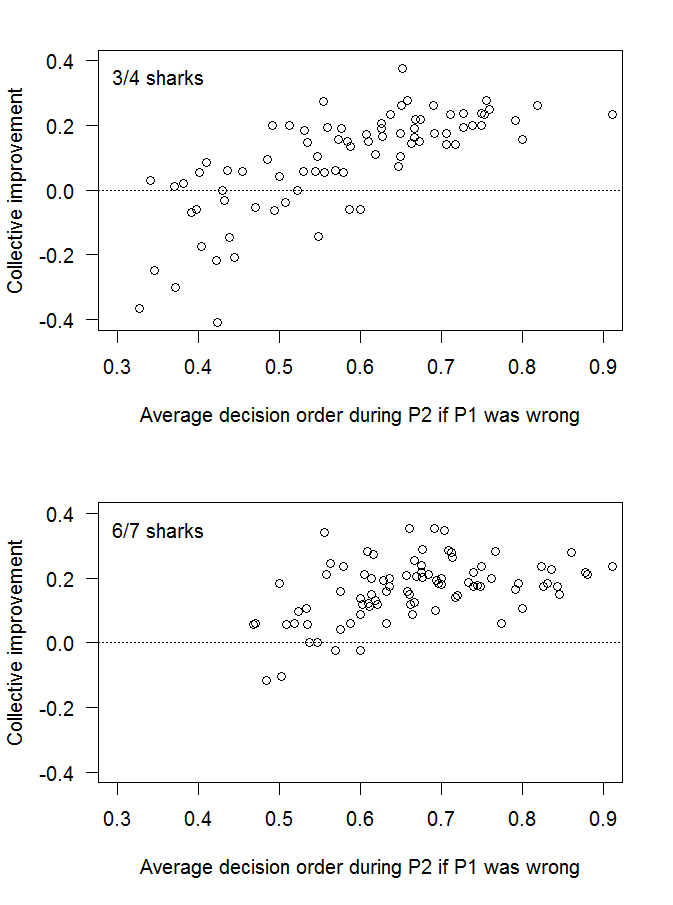

Supplement: ESM Supplementary Information [file rsos150222supp2.docx]
